# Genomics Recapitulates History in Europe

**DOI:** 10.1371/journal.pbio.1001556

**Published:** 2013-05-07

**Authors:** Robin Meadows

**Affiliations:** Freelance Science Writer, Fairfield, California, United States of America

[Fig pbio-1001556-g001]Most of us know our families back a few generations but, beyond that, have little idea who our ancestors were or where they lived. Jumping further back, all of us alive today likely share most of our ancestors from 3,000 to 4,000 years ago. What happened between then and now? We've pieced together a broad picture of human kinship based on disciplines from archeology to linguistics to history. In Europe, for example, several relatively recent migrations have helped shape links and gaps amongst today's populations. Now, in this issue of *PLOS Biology*, Peter Ralph and Graham Coop use genomic data to give us a closer look at the recent roots of modern Europeans.

**Figure pbio-1001556-g001:**
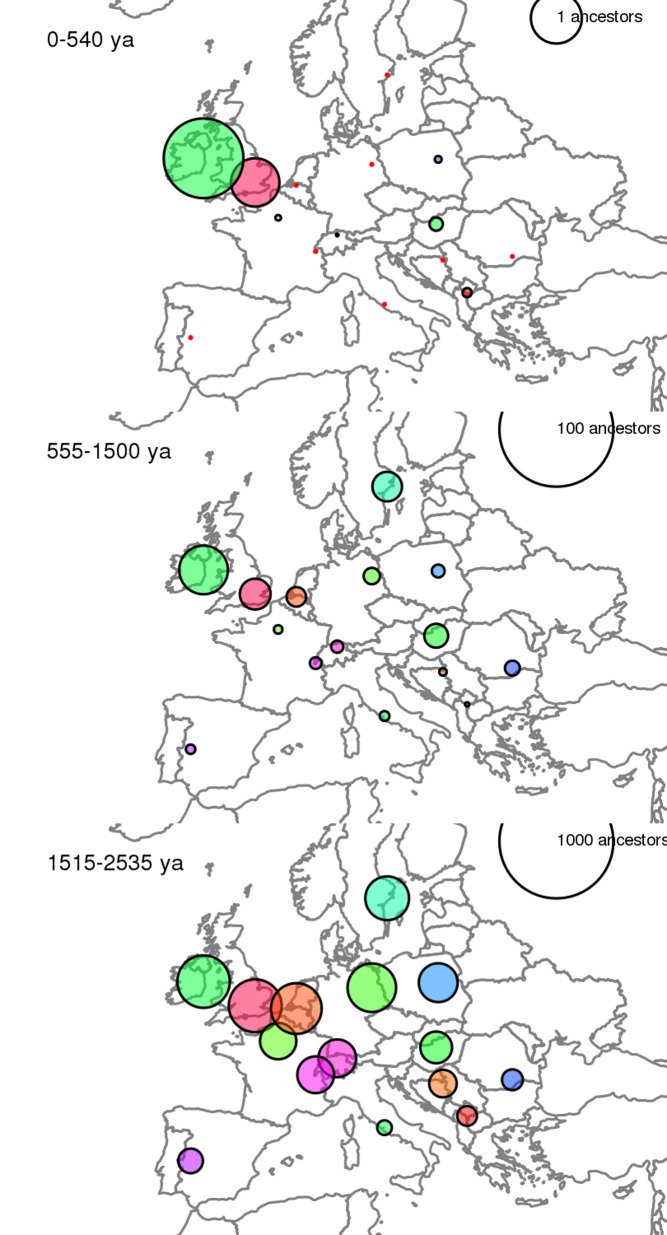
The distribution of distant cousins of modern-day people in the UK, at three different levels of geneological distance (circle size proportional to numbers of cousins; units are numbers of shared ancestors). Image credit: Peter Ralph and Graham Coop.

Previous work has shown that genotypes in Europe vary with latitude and longitude, and that genetic diversity tends to increase from north to south. To get a sharper picture of the interplay between recent relatedness and geography in Europe, Ralph and Coop compared genome-wide sequencing data from 2,257 people across the continent. The researchers used sharing of long genome segments between individual people as well as populations as a measure of common ancestry. Shared segments become progressively shorter back through history as they have undergone more generations of recombination. Thus, the longer a shared segment, the more recent the common ancestor.

As might be expected, comparison of these long shared segments by country typically showed that recent relatedness is highest amongst people who live near each other. There are, however, some noteworthy departures from this norm. People in the UK share more recent ancestors with people in Ireland than with others living in their own country. Likewise, people in Germany share more recent ancestors with the Polish than with other Germans. This pattern could reflect the migrations of smaller populations into a larger one.

Similarly, while recent relatedness generally drops evenly across geographic distances in Europe, a few exceptions stand out. Regardless of physical proximity, recent relatedness is low between the Italian peninsula and the rest of the continent. At the other end of the scale, recent relatedness is high within northern Europe as well as across eastern Europe – three times that within other regions at similar distances.

Ralph and Coop also used long shared segments to gauge how many recent ancestors are common to people across modern Europe, as well as roughly how long ago they lived. Because a person does not inherit genetic material from every single ancestor, this analysis only reveals a small fraction of the shared genealogical relationships; the researchers call this fraction “genetic common ancestors.”

The distributions of long segments revealed that genetic common ancestors from about 500 years ago are typically shared only by people who live in the same country today. Albanian speakers are at the high end, with about 90 genetic common ancestors within the last 500 years, and about 600 genetic common ancestors between the last 500 and 1,500 years . In contrast, just about any two people from almost anywhere across Europe today share hundreds of genetic ancestors from more than 1,500 years ago. The outliers are the Italian and Iberian (Spain and Portugal) peninsulas, where people have only about two genetic ancestors in common with populations elsewhere on the continent over the last 1,500 years.

Ralph and Coop then take into account that these genetic common ancestors are only a small fraction of the genealogical ancestors. Based on this, the researchers extrapolate that, conservatively, even people living in opposite ends of Europe today are likely to have a shared ancestry that includes everyone who both lived a thousand years ago and had descendants. The conclusion that all Europeans are related over such a short time period lends credence to the theory that everyone in the world is related over just the last few millennia. Indeed, the researchers speculate that Europe's common ancestors over the past millennium may also be shared worldwide.

Another intriguing finding is that the numbers and timing of common ancestors among different parts of Europe may reflect major events in the continent's history. Notably, the number of common ancestors within the last 1,000 to 2,000 years is particularly high within eastern Europe — similar to those in Ireland despite spanning far greater distances — and the timing fits with the series of migrations that began with the Huns in the 4th century and ended with the Slavs between the 6th and 10th centuries.

In support of linking this spike in common ancestry with these migrations, many of today's eastern Europeans who share long segments also speak Slavic languages. Furthermore, the regions with the fewest common ancestors (France, and the Italian and Iberian peninsulas) are also thought to have been largely untouched by the migrations of Huns and Slavs.

This work both corroborates and extends our understanding of Europe's recent past, adding another dimension to what other disciplines tell us about historical events. Besides giving us a fuller picture of the close ties between people around the world, delving into our recent past with population genomics could ultimately help answer these most basic of human questions: Where did we come from and how did we get here?

**Ralph P, Coop G (2013) The Geography of Recent Genetic Ancestry across Europe. doi:10.1371/journal.pbio.1001555**

